# Effect of the Nanoparticle Exposures on the Tomato Bacterial Wilt Disease Control by Modulating the Rhizosphere Bacterial Community

**DOI:** 10.3390/ijms23010414

**Published:** 2021-12-30

**Authors:** Hubiao Jiang, Luqiong Lv, Temoor Ahmed, Shaomin Jin, Muhammad Shahid, Muhammad Noman, Hosam-Eldin Hussein Osman, Yanli Wang, Guochang Sun, Xuqing Li, Bin Li

**Affiliations:** 1 State Key Laboratory of Rice Biology, Ministry of Agriculture Key Laboratory of Molecular Biology of Crop Pathogens and Insects, Key Laboratory of Biology of Crop Pathogens and Insects of Zhejiang Province, Institute of Biotechnology, Zhejiang University, Hangzhou 310058, China; 371112@zju.edu.cn (H.J.); 22016087@zju.edu.cn (L.L.); temoorahmed@zju.edu.cn (T.A.); 119287@zju.edu.cn (S.J.); nomansiddique834@gmail.com (M.N.); 2 Department of Bioinformatics and Biotechnology, Government College University, Faisalabad 38000, Pakistan; mshahid@gcuf.edu.pk; 3 Anatomy Department, College of Medicine, Taif University, P.O. Box 11099, Taif 21944, Saudi Arabia; h.hussein@tu.edu.sa; 4 State Key Laboratory for Managing Biotic and Chemical Threats to the Quality and Safety of Agro-Products, Zhejiang Academy of Agricultural Sciences, Hangzhou 310021, China; ylwang88@aliyun.com (Y.W.); sungc@mail.zaas.ac.cn (G.S.); 5 Hangzhou Academy of Agricultural Science, Hangzhou 310024, China

**Keywords:** 16S ribosomal RNA, antioxidants, microbiome, nanopesticides, nanotechnology

## Abstract

*Ralstonia Solanacearum* is one of the most infectious soil-borne bacterial plant pathogens, causing tomato bacterial wilt (TBW). Nanotechnology is an emerging area of research, particularly the application of nanoparticles (NPs) as nanopesticides to manage plant disease is gaining attention nowadays. However, the interaction between NPs and rhizosphere bacterial communities remains largely elusive. This study indicated that metal NPs (CuO, ZnO, and FeO) reduced the incidence of bacterial wilt to varying degrees and affected the composition and structure of the rhizosphere bacterial community. The results revealed that the application of metal oxide NPs can improve the morphological and physiological parameters of TBW infected tomato plants. Among all, CuONPs amendments significantly increase the Chao1 and Shannon index. In the early stage (the second week), it significantly reduces the relative abundance of pathogens. However, the relative abundance of beneficial *Streptomyces* bacteria increased significantly, negatively correlated with the relative abundance of pathogenic bacteria. In addition, the nano-treatment group will enrich some potential beneficial bacteria such as species from *Sphingomonadaceae*, *Rhizobiaceae*, etc. In general, our research provides evidence and strategies for preventing and controlling soil-borne disease tomato bacterial wilt with metal oxide NPs.

## 1. Introduction

Tomato (*Solanum lycopersicum*) is a major vegetable crop and a rich source of natural vitamins, essential amino acids, minerals, organic acids, and dietary fibers [1]. During the past few years, the tomato crop has been threatened by numerous bacterial and fungal phytopathogens [2]. Tomato bacterial wilt (TBW) is the most infectious soil-borne bacterial disease in the world. *Ralstonia solanacearum* is the causal agent of TBW and is recognized as a serious phytopathogen [3]. Over the last few decades, conventional chemical techniques involving pesticides and antibiotics have been used to treat plant diseases. However, chemical disease control requires capital investments and poses severe threats to environmental stability, human health, and agricultural production, leading to pathogen resistance [4]. Therefore, the production of tomatoes needs to be increased to meet future demands. Recently, the manipulation of the plant and soil microbiome has been considered an eco-friendly sustainable approach in plant disease management [5].

The rhizosphere is the zone of soil influenced by a plant root, which directly influences microbial growth and nutrient exchange. This root-zone supports microbial communities by providing nutritional substrates and microbes help in solubilizing the elemental form of nutrients for plant uptake [6]. Microorganisms attach to the plant root and facilitate nutrient exchange between root-to-plant systems [7]. Moreover, the plant growth-promoting rhizobacteria (PGPR) are a wide range of rhizosphere bacteria that actively inhabit the plants-root systems and increase crop growth by mobilizing nutrients, outcompeting soil-borne phytopathogens, and activating the plant physiological protection system [8]. Nanotechnology is an innovative research field with potential uses in the agriculture sector, partially in alleviating the environmental stresses by modifying the soil microbiome [9].

Recently, nano-enabled strategies have gained significant consideration due to their specific physical and chemical characteristics [10]. Previous research has reported that the application of metal oxide nanoparticles (NPs) as nanofertilizers significantly increases crop productivity and soil enzymatic activity and inhibits plant pathogens by modifying the function of soil microbiota [11,12]. Moreover, some research revealed that metal oxide NPs inhibit plant diseases by directly inhibiting pathogens and improving essential trace elements uptake [13,14]. For example, Servin et al. [15] revealed that metal oxide NPs significantly enhanced plant growth and suppressed disease by stimulating the antioxidant defense mechanisms in plants. Elmer and White [16] found that copper oxide nanoparticles (CuONPs) inhibited the *Verticillium* wilt disease of eggplant by 69% and significantly increased the plant biomass compared to the control. Kokina et al. [17] reported that the exposure of iron oxide nanoparticles (FeONPs) was able to stimulate the growth and physiological parameters of plants by inhibiting the soil pathogens. Xu et al. [11] observed that ZnONPs significantly increased the activity of different PGPR, such as *Cyanobacteria*, *Burkholderia*, and *Xanthomonas*. Ziaee and Ganji [18] revealed that SiO_2_ NPs inhibited the growth of pathogenic bacteria and enhanced the structural rigidity and strength of plants. However, there is a lack of systematic research on the prevention and control of soil-borne TBW disease through metal oxide NPs.

In order to develop effective nanopestcides for the control of TBW, the aim of the current study is to examine the in vivo antibacterial effect of metal oxide NPs (CuO, FeO, and ZnO) against *R. solanacearum* and their subsequent impacts on the soil bacterial community to reveal the ecological interaction of NPs with the microbiome in the soil-plant system.

## 2. Results

### 2.1. Characterization of Metal Oxide NPs

**Figure 1:**
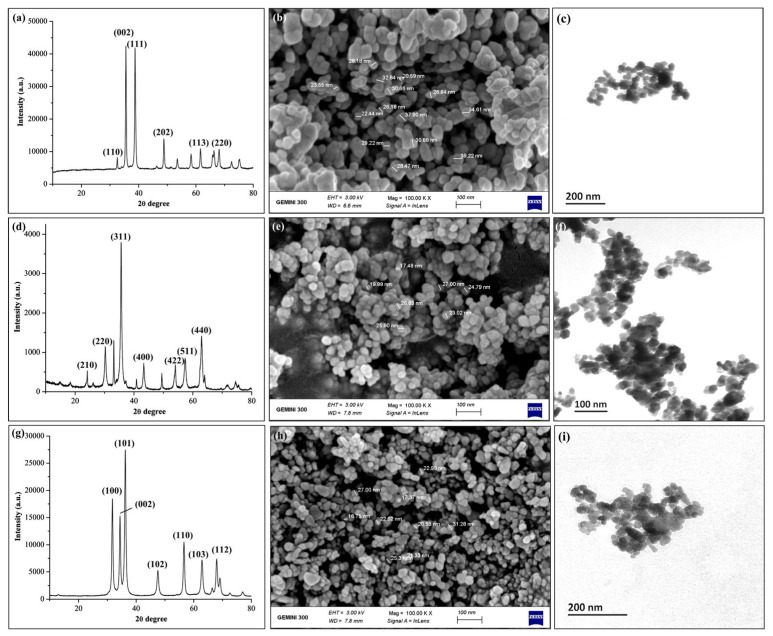
Characterization of three metal oxide nanoparticles (FeO, ZnO and CuO) using XRD spectral analysis (**a**) FeO, (**d**) ZnO and (**g**) CuO. Scanning electron micrographs (**b**) FeO, (**e**) ZnO and (**h**) CuO [scale bar = 100 nm]. Transmission electron micrographs (**c**) FeO, (**f**) ZnO and (**i**) CuO [scale bar = 100–200 nm].

The crystalline structure of three metal oxide NPs was determined based on the XRD spectral analysis (Figure 1). In the present study, the XRD spectra of CuONPs revealed the typical diffraction peaks at 32.4, 35.5, 38.7, 48.9, 61.5, and 68.1, which corresponded to (110), (002), (111), (202), (113), (220) crystal planes, respectively. Similarly, FeONPs showed diffraction peaks at (24.2), (30.2), (35.6), (43.31), (54.1), (57.3) and (62.9), which corresponds to crystal planes of (210, 220, 311, 400, 422, 511 and 440), respectively. In the case of ZnONPs, XRD spectra depicted characteristic peaks at (31.6), (34.4), (36.2), (47.5), (56.6), (62.8), and (67.9) were indexed to the standard planes of (100, 002, 101, 102, 110, 103 and 112), respectively. Moreover, the surface morphology and particle size of metal oxide NPs were observed by SEM and TEM analyses. In the current study, electron microscopy images showed that the metal oxide NPs have spherical shapes with sizes ranging from 20–39 nm for CuONPs, 19–27 nm for FeO NPs and 16–31 nm for ZnONPs (Figure 1). The average hydrodynamic size of metal oxide NPs were shown in (Table S1).

### 2.2. Effect of Metal Oxide NPs on the TBW Infected Plant Morpho-Physiological Parameters

**Figure 2:**
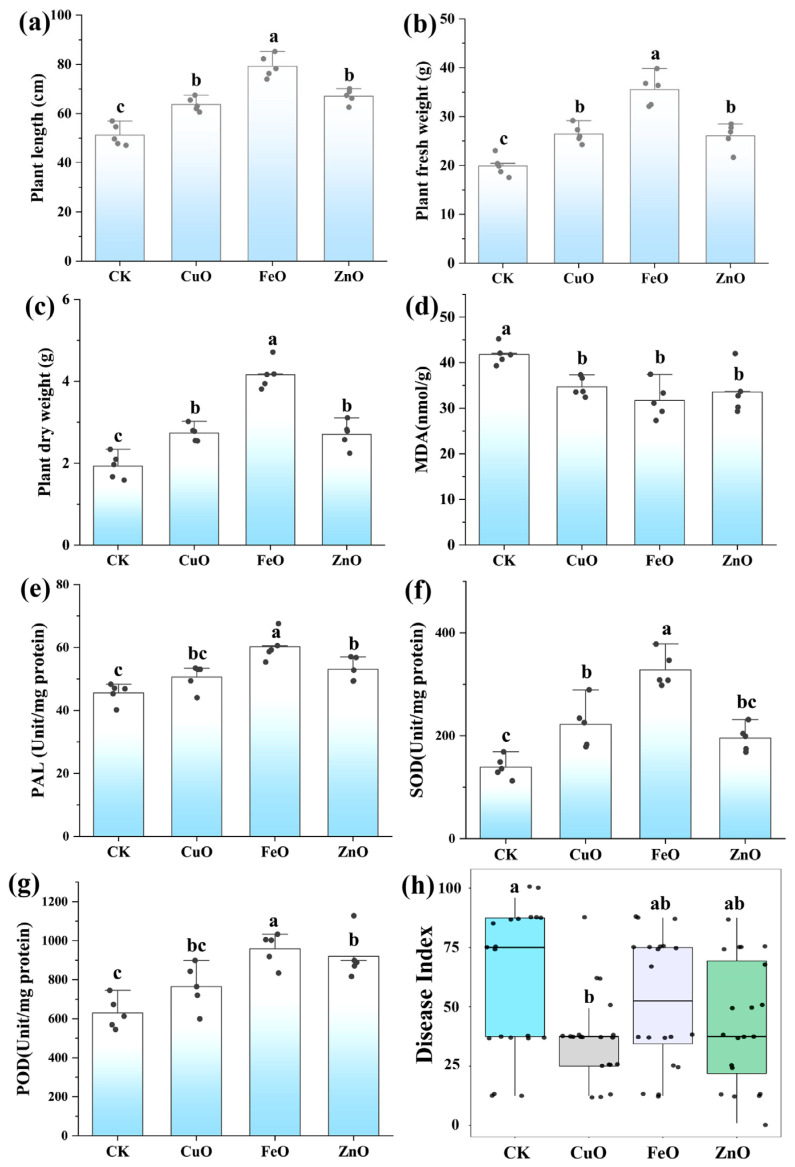
Effect of three metal oxide nanoparticles (CuO, FeO and ZnO) on the TBW infected plant growth, physiology, and disease index. (**a**) Plant height, (**b**) Plant fresh weight, (**c**) Plant dry weight, (**d**) Malondialdehyde (MDA), (**e**) Phenylalanine ammonia lyase (PAL), (**f**) Superoxide dismutase (SOD), (**g**) Peroxidase and (**h**) Disease index. Different letters reveal the significance among different treatments (*p* < 0.05).

Results indicated that the three metal oxide NPs significantly improved the TBW infected plants’ growth (*viz.*, length, fresh, and dry weights) by mitigating the effects of TBW disease compared to the control treatment (Figure 2). The application of CuONPs, FeONPs, and ZnONPs caused 24.3%, 54.6%, and 30.8% increases (Figure 2a), respectively, in tomato plant length, compared to the corresponding diseased control. Similarly, TBW infected plants treated with three metal oxide NPs (CuO, FeO, and ZnO) resulted in 32.8%, 78.3%, and 30.6% increases in fresh weight (Figure 2b), and 41.0%, 54.1%, and 40.0% increase in dry weight (Figure 2c), respectively, compared to the control. Among three metal oxide NPs, FeONPs showed the maximum growth-promoting effect on diseased tomato plants.

A significant increase in the cellular antioxidative enzymes and reduction in ROS production was observed in metal oxide NPs treated TBW infected plants compared with non-treated TBW infected control plants (Figure 2). The metal oxide NPs treatments reduced ROS (MDA) contents by 16.9% (CuONPs), 24.1% (FeONPs) and 19.6% (ZnONPs) as compared with non-treated TBW infected control plants (Figure 2d). After the treatment with CuONPs, FeONPs, and ZnONPs, tomato plants showed 21.5%, 52.3%, and 46.3% increase in POD contents (Figure 2g), respectively, as compared with non-treated TBW infected control plants. Likewise, 59.6%, 35.6%, and 40.5% increases were observed in the SOD contents of tomato plants treated with CuONPs, FeONPs, and ZnONPs (Figure 2f), respectively, in contrast to the control group. Similarly, soil application of CuO, FeO, and ZnONPs significantly increased the PAL contents by 11.6%, 32.35%, and 16.5%, respectively, as compared with corresponding diseased control (Figure 2e). However, the treatment with FeONPs showed the highest impact on antioxidative enzyme activity compared to other NPs.

### 2.3. Effect of Metal Oxide NPs on the Disease Incidence

The disease incidence of different treatment groups is shown in Figure 2h. The average disease incidence of the control group is 60.5%. The application of the CuONPs treatment decreased the disease incidence significantly to 36.9%, while the disease incidence in the case of treatment group FeONPs and ZnONPs was not decreased significantly and it was counted as 52.8% and 42.8%, respectively, compared to the control plants. The above information showed that CuONPs significantly reduced tomato bacterial wilt disease, while FeONPs and ZnONPs application could not reduce the disease occurrence significantly, and their ultimate impact was much less as compared CuONPs.

### 2.4. Microbial Alpha Diversity and Beta Diversity

**Figure 3:**
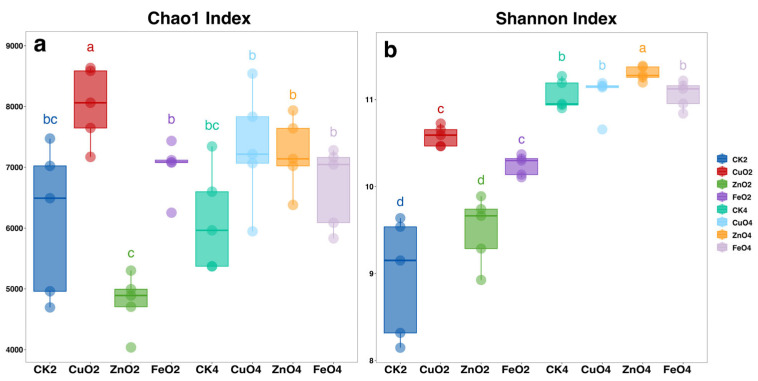
Chao1 (**a**), Shannon (**b**) index under the exposure of CK, CuO, FeO, and ZnO NPs. Different letters reveal the significance among different treatments (*p* < 0.05). The horizontal bars within boxes represent medians. The tops and bottoms of boxes represent the 75th and 25th percentiles, respectively. The upper and lower whiskers extend to data no more than 1.5× the interquartile range from the upper edge and lower edge of the box, respectively. Abbreviation: second week of nanoparticles treatment (Control_2_, CuO_2_, ZnO_2_, and FeO_2_), fourth week of nanoparticles treatment (Control_4_, CuO_4_, ZnO_4_, and FeO_4_).

It is well established that the Chao1 and Shannon indexes represent the richness and diversity of microbial communities, respectively. After the second week, the Chao1 index was significantly increased in the CuONPs treatment group (*p* < 0.05), and the ZnONPs treatment significantly decreased (*p* < 0.05), while the FeONPs index had no significant change (*p* > 0.05). The Chao1 index of all treatment groups had no significant difference in the fourth week (Figure 3a).

In addition, CuONPs and FeONPs significantly increased the Shannon index in the second week (Figure 3b), while ZnONPs had no significant difference. In the fourth week, only ZnONPs significantly increased the Shannon index (*p* < 0.05). Interestingly, the Shannon index showed a significant increase overall (*p* < 0.05) compared with the second week. The above results indicate that in the early stage of treatment (the second week), NPs can significantly change the richness and diversity of rhizosphere microbial communities, of which CuONPs increase most significantly. Eventually, the impact of NPs on the diversity of bacterial communities will tend to disappear.

**Figure 4:**
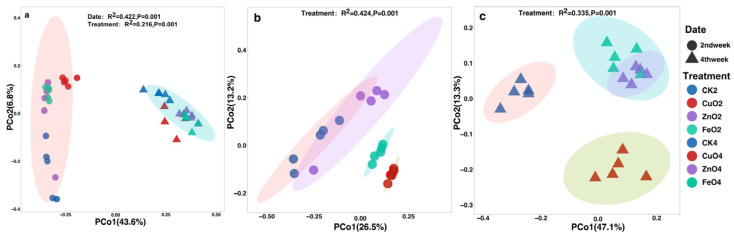
PCoA (principal coordinates PCo1 and PCo2) analysis of the bacterial communities based on the Bray–Curtis distance (**a**) PCoA analysis of all samples, (**b**) PCoA analysis of samples in the second week (**c**) PCoA analysis of samples in the fourth week (*p* < 0.05, permutational multivariate analysis of variance (PERMANOVA)). Abbreviation: 2nd week of nanoparticles treatment (Control_2_, CuO_2_, ZnO_2_, and FeO_2_), 4th week of nanoparticles treatment (Control_4_, CuO_4_, ZnO_4_, and FeO_4_).

We performed a principal coordinate PCOA analysis based on the Bray–Curtis distance to further compare the effects of NPs on the rhizosphere microbial community. The PCoA analysis showed that the soil rhizosphere microbial community formed two different clusters. The samples of the second week and the fourth week were separated along the first axis (PERMANOVA, *p* < 0.05). The first axis explains 43.6% of the overall variation, and the second axis explains 6.8% (Figure 4a). We perform ADONIS (a nonparametric multivariate analysis of variance) on all samples to determine the effect size of different NPs and sampling times. When all the samples were analyzed together, we found that the sampling time explained 42.2% of the variation (*p* = 0.001), while the different NPs explained 21.6% of the variation (*p* = 0.001) (Figure 4a). It shows that sampling time has a greater impact on the total microbial community than the different NPs. Moreover, we divided the samples according to different sampling time points for analysis (Figure 4b,c). ADONIS analysis showed that the different NPs treatment groups accounted for 42.4% of the sampling variation (*p* = 0.001) at the second week. In the fourth week, the different NPs treatment groups explained 33.5% of the variation (*p* = 0.001). It shows that the different NPs treatments in the second week have a greater impact on the bacterial community as compared with the fourth week. However, different NPs have different effects on the rhizosphere microbial community, and the earlier (second week) impact on the plant rhizosphere microbial community is greater than the later (fourth week).

### 2.5. Effect of Metal Oxide NPs on Bacterial Community Diversity

**Figure 5:**
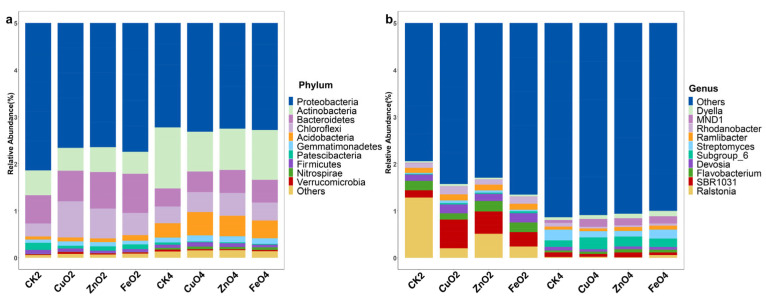
Relative abundance distribution at the level of microbial phylum-level (**a**) and genus-level (**b**) in the rhizosphere. Abbreviation: 2nd week of nanoparticles treatment (CK2, CuO_2_, ZnO_2_, and FeO_2_), 4th week of nanoparticles treatment (CK4, CuO_4_, ZnO_4_, and FeO_4_).

The original data were quality-controlled, and a total of 2,506,503 high-quality 16S rRNA gene sequences were obtained from all 40 rhizosphere samples. Among them, the high-quality sequences of each sample range from 38,725 to 85,286. A total of 16,894 bacterial ASVs were identified. After NPs treatment, the bacterial community structure is mainly composed of *Proteobacteria*, *Actinobacteria*, *Bacteroidetes*, *Chloroflexi*, *Acidobacteria*, *Gemmatimonadetes* at the phylum level (Figure 5a). In the second week, the relative abundance of *Proteobacteria* and *Patescibacteria* in the NPs treatment group was significantly reduced (*p* < 0.05) compared with the control CK2. The relative abundance of *Chloroflexi* increased significantly (*p* < 0.05), and there was no significant difference in bacterial community composition among different NPs. In addition, compared with the control group (CK4), the relative abundance of *Actinobacteria* was significantly lower in the fourth week (*p* < 0.05). Interestingly, the relative abundance of *Ralstonia* spp. in the NPs treatment group decreased significantly in the second week as compared with the respective control (Figure 5b and S1). At the same time, there was no difference in the fourth week. In addition, the relative abundance of CuONPs in the second week of Streptomyces was significantly higher than that of the control (CK2) (wilcox. test, *p* < 0.05). Spearman correlation analysis showed that Streptomyces and *Ralstonia* spp. were negatively correlated (R = −0.593, *p* = 0.006). The above results indicate that NPs could affect the abundance of bacteria in the rhizosphere soil to regulate the composition of the bacterial community.

### 2.6. Differences in the Rhizosphere Microbiome and Biomarker

**Figure 6:**
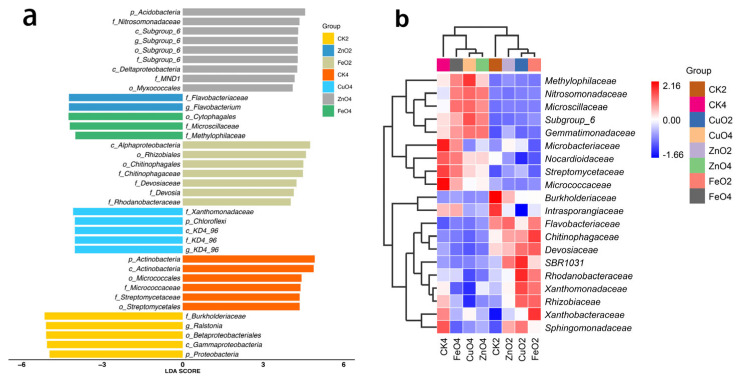
Linear discriminant analysis (LDA) effect size (Lefse) of the bacterial taxa (**a**), which identifies the most differentially abundant taxa among the different nanoparticles treatment. Only taxa with LDA values greater than 4 (*p* < 0.05) are shown. Hierarchical clustering analysis and heat map at the (**b**) family level. The tree plot represents a clustering analysis of the top 20 bacteria at family levels according to their Pearson correlation coefficient matrix and relative abundance, the upper tree plot represents a clustering analysis of soil samples according to an euclidean distance of data. Abbreviation: 2nd week of nanoparticles treatment (CK2, CuO_2_, ZnO_2_, and FeO_2_), 4th week of nanoparticles treatment (CK4, CuO_4_, ZnO_4_, and FeO_4_).

We used linear discriminant analysis of effect size (Lefse) (LDA > 4, *p* < 0.05) to reveal the biomarkers with the largest difference in rhizosphere soil microbial communities under different NPs treatment conditions. A total of 14 biomarkers were found in the second week (Figure 6a), and the control (CK2) mainly identified five types *Burkholderiaceae*, *Ralstonia*, *Betaproteobacteriales*, *Gammaproteobacteria*, and *Proteobacteria*. FeO_2_ is enriched with *Alphaproteobacteria*, *Rhizobiales*, *Chitinophagales*, *Chitinophagaceae*, *Devosiaceae*, *Devosia*, *Rhodanobacteraceae*, while ZnO_2_ is rich in *Flavobacterium* and *Flavobacteriaceae.* In the fourth week, a total of 23 biomarkers were identified in all treatments. Among them, the relative abundance of the six bacteria *p_Actinobacteria*, o_Actinobacteria, *Micrococcales, Micrococcaceae, Streptomycetales*, and Streptomycetales in the control group CK4 was higher. Three bacteria, *Xanthomonadaceae, Chloroflexi*, and KD4_96 were enriched in the CuO4 treatment group and ZnO4 was enriched in *Acidobacteria, Nitrosomonadaceae, Subgroup_6*, *Deltaproteobacteria, MND1, Myxococcales.* The FeO4 treatment group was enriched in *Cytophagales, Microscillaceae,* and *Methylophilaceae.* We further visually explained the difference in relative abundance composition (family level) of the rhizosphere microbial community under nano-treatment conditions through heat maps (Figure 6b). In the second week, the nano-treatment enriched *Sphingomonadaceae, Rhizobiaceae, Rhodanobacteraceae, Xanthomonadaceae*, and SBR1031 as a whole (*p* < 0.05). *Intrasporangiaceae, Nocardioidaceae,* and *Burkholderiaceae* were significantly reduced (*p* < 0.05). However, the nano-treatment group was significantly enriched in *Nitrosomonadaceae, Methylophilaceae, Microscillaceae, Gemmatimonadaceae, Subgroup_6* (*p* < 0.05) at the fourth week. The results demonstrate that NPs can reduce or increase the existence of certain specific species that change the community structure of bacteria in rhizosphere soil.

### 2.7. Co-Occurrence Networks Analysis

**Figure 7:**
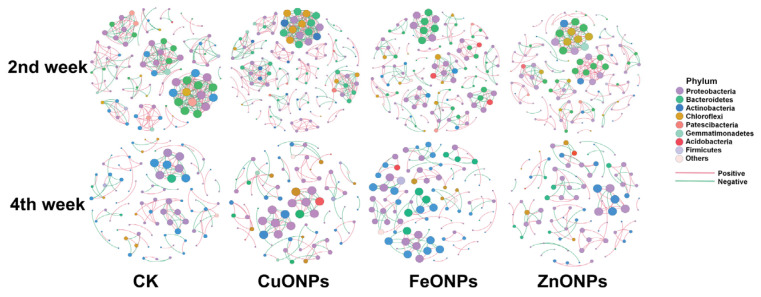
Effect of three metal oxide nanoparticles (CuO, FeO, and ZnO) on the co-occurrence patterns of soil bacterial community. Networks were constructed at the amplicon sequence variants (ASV) level. The size of nodes (ASVs) represents the relative abundance of microbes, and the nodes are colored according to phylum. Green lines and red lines represent negative correlation and positive correlation.

We conducted a co-occurrence network analysis to explore the complexity of connections between rhizosphere soil microbial communities in different treatment groups. Moreover, we calculated the topological properties of the co-occurrence network to characterize the differences between different groups (Figure 7 and Table S2). The CK2 microbial network of the control group consists of 105 nodes and 305 edges (168 positive edges, 137 negative edges, an average degree was 5.81), with a modularity of 0.822. The CuONPs, FeONPs, and ZnONPs are 384 (positive: 231, negative: 153, average degree 6.371), 226 (positive:125, negative:101, average degree 3.348), 244 (positive: 166, negative: 78, average degree 3.904) consisting of edges and 143,135,125 nodes, respectively. The modularity was 0.839, 0.935, 0.873, respectively.

In the fourth week, the microbial network of the control group consisted of 96 nodes and 115 edges (62 positive edges, 53 negative edges, an average degree was 2.396), with a modularity of 0.905. The CuONPs, FeONPs, and ZnONPs are respectively composed of 115 (positive: 61, negative: 56, average degree 2.753), 117 (positive: 61, negative: 55, average degree 2.32), 116 (positive: 58. negative: 42, average degree 2.083) consisting of edges and 85,100,96 nodes. The modularity is 0.91, 0.948, 0.943, respectively.

## 3. Discussion

Over the last few decades, the continued application of metals, especially Cu as an antimicrobial agent in either salt or bulk hydroxide form, will contribute to soil contamination and accumulation as well as potentially lead to resistance in target phytopathogens [19]. Therefore, novel and sustainable food-production strategies are sorely needed. Recently, nano-enabled agrichemicals have attracted significant attention due to the controlled release of micronutrients and activate plant defense systems to suppress disease. Previous studies showed that the levels of Cu added in CuONPs treatments are an order of magnitude less than that in conventional Cu-containing fungicide treatments, but equivalent or enhanced efficacy is achieved [19,20]. However, metallic-based nanomaterials exhibit promising potential for plant disease control by modulating the plant nutrition and host defense activation [19,20]. This study explored the control effects of three metal oxide NPs (FeO, CuO, and ZnO) against tomato bacterial wilt (TBW) disease and their subsequent impact on rhizosphere microbial community shift. Moreover, our study revealed that these NPs significantly reduced the incidence of TBW by improving the infected plant biomass, antioxidant enzyme activity and decreasing the reactive oxygen species concentration with the maximum impact being observed by CuONPs. In the literature, several studies have reported the antibacterial potential of metal oxide NPs against phytopathogens [13,16]. However, the systematic optimization of NPs dose and their subsequent interaction with soil microbial communities is largely unknown.

In a recent study, Parveen et al. [21] reported that the foliar exposure of ZnONPs reduced the TBW disease indices by improving the growth, photosynthetic pigments, proline contents in tomato plants. Similarly, Chen et al. [22] also observed the antibacterial efficacy of CuONPs against bacterial wilt pathogen *R. solanacearum.* Moreover, previous studies have also shown that the use of metal oxide NPs was able to increase tomato plant growth and antioxidative enzymes activity [23,24]. For example, Faizan et al. [25] observed that ZnONPs supplementation significantly improved tomato plant growth, the activity of antioxidant enzymes under stress conditions. Faizan et al. [26] reported that ZnONPs applied through root dipping increased the tomato plant growth and antioxidant enzyme concentrations.

We measured the microbial community diversity in the tomato rhizosphere with three kinds of NPs (CuONPs, ZnONPs, FeONPs) using 16S rRNA gene high-throughput sequencing. The diversity of microbial communities is crucial to the soil ecosystem’s integrity and ecological function [27]. We used the alpha diversity index (Chao1 and Shannon index) to measure the bacterial diversity at the ASV level under different nano-treatment conditions (Figure 3a,b). The results showed that CuONPs significantly increased the Chao1 and Shannon indexes in the second week after nano-treatment. ZnONPs reduced the Chao1 index but had no significant effect on the Shannon index. FeONPs significantly increased the Shannon index and did not affect the Chao1 index. In the fourth week, the nano-treatment group had no significant effect on the Chao1 index, and only ZnONPs increased the Shannon index. We considered that metal oxide NPs could affect community diversity, and CuONPs had the most obvious effect on increasing the richness and diversity of microbial communities in the short term and will tend to disappear in the fourth week. It is worth noting that treatment with ZnONPs could reduce the richness of the microbial community in the short term, but over time, it would gradually return to the normal community structure and increase the diversity of the microbial community. Fang et al. [28] found that the application of FeONPs has a positive impact on the soil microbial community. In general, the effects of NPs on the richness (Chao1) and diversity (Shannon) of the rhizosphere microbial community are different due to different NPs, and the early impact on the rhizosphere microbial community is greater than the later impact (Figure 3). PCoA results showed that the rhizosphere microbial communities were separated according to different sampling times and different treatments (Figure 4a, PERMANOVA, *p* < 0.05), and overall, the sampling time explained 42.2% (*p* = 0.001) of the variation. NPs treatment showed 21.6% (*p* = 0.001) variation. We then split all samples by sampling times to analyze them separately (Figure 4b,c). NP-treatment showed 42.4% variation in the second week (Figure 4b, PERMANOVA, *p* = 0.001), and explained 33.5% of the variation in the fourth week (Figure 4c, PERMANOVA, *p* = 0.001). Therefore, there were significant differences in bacterial community composition at different sampling times and between different nano-treatments. In addition, CuONPs would significantly increase the diversity and richness of the microbial community in the short term and enhance the tolerance of tomato rhizosphere microbial communities to external biological stresses, and CuONPs could significantly reduce disease incidence (Figure 2h). Therefore, we believed that metal oxide NPs (especially CuONPs) application induces soil suppressiveness against TBW disease by reshaping the rhizosphere microbial community.

At the phylum level, all samples included Proteobacteria, Actinobacteria, Bacteroidetes, Chloroflexi, and Acidobacteria in five phyla (Figure 5a), which is consistent with the results of Liu et al. [29]. Compared with the control (relative abundance of 25.6%), the relative abundance of *Ralstonia* spp. in the three nano-treatment groups decreased to 4.0%, 10.1%, and 4.8% (Figure 5b and Figure S1). In addition, the relative abundance of Streptomyces in the CuONPs treatment group increased significantly (*p* < 0.05). It shows that NPs could reduce the content of pathogenic bacteria and enrich some specific beneficial bacteria. Reducing the number of pathogenic bacteria has a good inhibitory effect on tomato bacterial wilt [30]. Streptomyces can produce antibacterial compounds, resist environmental disturbances through thick-walled spores, and is potentially beneficial bacteria [31]. To further explore the impact of nano-treatment on microbial groups, we used linear discriminant analysis of the effect size (Lefse, LDA > 4). The control group *Ralstonia* spp. was significantly enriched in the second week, while ZnONPs, FeONPs, and CuONPs were not identified (Figure 6a). In the fourth week, *Ralstonia* spp. was not identified in all treatments. It shows that NPs could reduce the relative abundance of *R. solanacearum* in tomato rhizosphere soil. Furthermore, we could know from the heat map that the nano-treatment group was significantly enriched in *Sphingomonadaceae, Rhizobiaceae, Rhodanobacteraceae, Xanthomonadaceae*, and other bacteria in the second week (Figure 6b). *Nitrosomonadaceae, Methylophilaceae, Microscillaceae, Gemmatimonadaceae*, etc. were significantly enriched in the fourth week. All of the families above belong to Proteobacteria. Proteobacteria are important companion bacteria of plants, which are beneficial to resist life or abiotic stress [32]. For example, it is worth noting that *Rhizobiaceae* is a potentially beneficial bacterium that plays an important role in promoting plant growth and resisting external stress [33,34]. *Sphingomonadaceae* were producers of extracellular polysaccharides, which were beneficial to the development of soil aggregates [35]. Although the response mechanism of *Sphingomonadaceae* and *Rhizobiaceae* to NPs needs further study. However, these results indicate that NPs were likely to change the bacterial composition of the tomato rhizosphere microbial community by recruiting certain specific bacteria to reduce the occurrence of diseases.

Co-occurrence networks could provide a new perspective for microbial interaction analysis. Here, we apply correlation-based network results to analyze the interaction between ASVs of different treatments (Spearman’s r > 0.7 or r < −0.7, *p* value < 0.01) (Figure 7 and Table S2). For example, the number of network nodes and edges in the fourth week decreases, the average degree decreases, but the modularity increases as compared with the second week. The CuONPs treatment group showed a higher average degree (6.371) and modularity (0.839) in the second week, which indicates that the community after CuONPs treatment is more complicated, and the diversity of microflora shows higher microbial interactions and stronger niche competition [36,37]. The average degree value describes the level of connectivity between ASVs.

Overall, our results show that the ASV of CuONPs in the nano-treatment was more closely related than the control, and this connection is more positive. The microbial community structure is more diverse and compact and has a higher tolerance to abiotic and biotic stress [38]. Therefore, we speculate that the close relationship between the bacterial communities in the CuONPs treatment group may help to improve the resistance to bacterial wilt stress. In the second week, ZnONPs and FeONPs showed higher modularity (0.873 for ZnONPs and 0.935 for FeONPs) and a lower average degree (3.904 for ZnONPs and 3.348 for FeONPs) than the control. It may indicate that microbial communities respond more quickly to environmental disturbances [39]. In addition, all treatment groups gradually stabilized in the fourth week (Table S2). The above results revealed that metal oxide NPs could affect the interaction between bacteria in the rhizosphere microbial community. We believe that these may play an important role in reducing the occurrence of diseases.

## 4. Materials and Methods

### 4.1. Characterization of Nanoparticles

In this study, three different metal oxide NPs (CuO, FeO, and ZnO) with 98% purity were purchased from (Xuzhou Jiechuang, Material Technology Co., Shanghai, China). The crystalline nature of three metal oxide NPs was observed by using an X-ray diffractometer (Siemens-D5000, Munich, Germany). The particle size and surface morphology of NPs were determined using transmission (TEM, JEM1230, JEOL Ltd., Tokyo, Japan) and scanning (Gemini-SEM300, Carl Zeiss AG, Jena, Germany) electron microscopy. The suspension of NPs was sonicated (KQ-300DE Kunshan Ultrasonic Instrument Co., Ltd., Jiangsu, China). Afterward, the NPs size distribution in a water suspension was observed through Zetasizer (NanoZS90, Malvern, UK) [13].

### 4.2. Pot Experiment

#### 4.2.1. Plant Material and Experimental Design

The soil used in this experiment was collected from the agricultural field of the Zijingang campus of Zhejiang University, China (120°08′ E and 30°30′ N). Before further analysis, the samples were air-dried, crushed, and sieved with a 3-mm mesh. Tomato seeds (cv. 99 Hezuo 903) were sterilized using 5% sodium hypochlorite for five minutes and washed three times with distilled water. The sterilized tomato seeds were put in petri plates for germination on wet filter paper (no. 1) in the dark conditions at 28 °C for 3 days. Afterward, 5 kg soil was added in pots followed by planting tomato seeds and placed in a greenhouse (27–30 °C day/night temperature, 50% humidity with 14 h light/10 h dark cycles). At the 3-leaf stage, 5 mL of *R. solanacearum* culture (10^7^ cfu mL^−1^) was used to inoculate the roots of tomato seedlings by irrigating the roots. Each treatment group contains five replicates and each replicate contains five tomato plants (a total of 25 plants per NPs or control treatment, resulting in 100 plants). The soil in each pot was supplemented with water suspensions of three different metal oxide NPs at the concentration of 500 mg kg^−1^ soil, which was carried out according to the method [40]. The control pots were supplemented with sterile distilled water without NPs. After the 2nd and 4th weeks of inoculation, the rhizosphere soil samples were collected for DNA extraction and disease severity was evaluated based on the leaf wilt ratio as the average value of the disease index for each plant using a scale of 0–4 (0: no visible symptoms; 1: 25% of leaves wilting; 2: 50% of leaves wilting; 3: 75% of leaves wilting; and 4: 100% of leaves wilting).

#### 4.2.2. Determination of Plant Growth and Physiological Parameters

In order to evaluate the influence of NPs on plant growth and development, tomato seedlings were uprooted and washed two times with deionized water after 4 weeks of pathogen inoculation. The length of roots and shoots was calculated by using a measuring scale. Moreover, the fresh and dry weight of roots and shoots were determined on an electronic balance. Each of the above treatment groups contains five replicates, and each replicate contains five tomato plants. We take the average of the physiological indicators of five tomatoes as a replicate. The tomato leaf tissue samples were used for the measurement of malondialdehyde (MDA) concentration according to the detection kit protocol (Grace Biotechnology Co., Ltd., Suzhou, China) [41]. The samples (0.5 g) were ground with 0.2% trichloroacetic acid and centrifuged at 4 °C, 12,000 rpm for 10 min. A total of 200 μL supernatant was added in 300 μL of kit solution and heated in a water bath at 90 °C for 35 min. Afterward, the reaction mixture was centrifuged again at 4 °C, 12,000 rpm for 10 min, and the supernatant absorbance was measured at 532 nm and 600 nm using a microplate reader (SpectraMax 190, Temecula, CA, USA). For estimation of antioxidant contents, phenylalanine ammonia-lyase (PAL), peroxidase (POD), polyphenol oxidase activity (PPO), and superoxide dismutase (SOD) concentrations were analyzed with antioxidant assay kits (Suzhou Grace Biotechnology Co., Ltd., Suzhou, China). The antioxidant enzymes activity was measured by a microplate reader (SpectraMax 190, Temecula, CA, USA) at 450, 470, 290, 420 nm, respectively, according to Chance and Maehly [42].

### 4.3. 16S rRNA Amplicon Sequencing Analysis

#### 4.3.1. Collection of Rhizosphere Samples and DNA Extraction

The tomato rhizosphere soil was sampled in the second and fourth weeks after inoculation. In the second week, five tomato plants (one plant from one replicate) were sampled for each treatment. In the fourth week, we mixed the remaining four tomatoes (in each replicate) rhizosphere soil samples of each treatment. We gently uprooted the plant from the pot for rhizosphere soil samples, removed the extra soil from tomato plant roots by gentle shaking, and then collected the rhizosphere soil samples associated with the root system. Afterward, the extraction of total genomic DNA from samples was carried out according to the manufacturer’s instructions using the OMEGA Soil DNA isolation Kit (Omega, Bio-Tek, Norcross, Norcross, GA, USA). The quality of extracted DNA was determined by using a NanoDrop (ND-1000) spectrophotometer (Thermo Fisher Scientific, Waltham, MA, USA).

#### 4.3.2. 16S rRNA Amplicon Sequencing

The PCR amplification for the V3–V4 region of tomato rhizosphere bacterial 16S rRNA genes was carried out using the universal forward primer 338F (5′-ACTCCTACGGGAGGCAGCA-3′) and the universal reverse primer 806R (5′-GGACTACHVGGGTWTCTAAT-3′) [43]. Multiplex sequencing was carried out by incorporation of 7 bp sample-specific barcodes into the primers. The components of the PCR included 14.75 μL of ddH_2_O, 0.25 μL of Fast pfu DNA Polymerase (5 U/μL), 5 μL of buffer (5×), 2 μL (2.5 mM) of dNTPs, 1 μL of DNA Template, 1 μL (10 uM) of each Forward and Reverse primer. The PCR amplicons were purified with V azyme V AHTSTM DNA clean beads (Vazyme, Nanjinng, China). Afterward, amplicons with equal amounts were pooled and 2 × 250 bp pair-end sequencing was accomplished through the Illumina MiSeq system (Shanghai Personal Biotechnology Co., Ltd., Shanghai, China).

### 4.4. Bioinformatics and Statistical Analysis

The bioinformatics analysis of the microbiome was accomplished using QIIME2 2019.4 according to tutorials (https://deocs.qiim2.org/2019.4/tutorials/, accessed on (25 July 2020) with slight modifications [44]. In brief, sequence raw data were demultiplexed with the demux plugin, then primers were trimmed with the cutadapt tool. By using the DADA2 plugin, the sequence was filtered, combined, denoised, and removed the chimera according to [45]. The alpha- and beta-diversity metrics were assessed by using the diversity plugin. The feature-classifier plugin was used to assign taxonomy to amplicon sequence variants (ASVs) using a classify-sklearn naïve Bayes taxonomic classifier against the SILVA Release 132 Database [46].

The sequence data were analyzed by using QIIME2 and R packages (v3.6.0). The alpha diversity indices, including Shannon and Chao1 index, were estimated with the ASV table in QIIME2, and then visualized in the box plots. The analysis of beta diversity was carried out to observe the structural variation of rhizosphere soil microbiome across samples with Bray-Curtis metrics, principal coordinate analysis (PCoA) [47]. The difference of rhizosphere soil microbiota between groups was determined by analysis of similarities (ANOSIM), Permdisp using QIIME2, and Permutational multivariate analysis of variance (PERMANOVA). Linear discriminant analysis effect size (LEfSe) was carried out by using default parameters to observe the differentially abundant taxa between groups [48]. To investigate the impact of NPs on bacterial co-occurrence patterns, a Spearman correlation matrix among tomato rhizosphere soil microbial community was measured through “igraph” and “hmisc” packages based on the relative abundance of ASVs at different treatments (CK, CuONPs, FeONPs, and ZnONPs). The high relative abundances (RA > 1%) and statistically significant correlations (*p* < 0.01, Spearman’s coefficient N > 0.7 or < −0.7) among ASV levels were built-in into the network analysis. In order to observe the topology of the co-occurrence networks, the network graphs analysis was performed based on these measurements including average degree, Modularity, edges, and nodes.

## 5. Conclusions

In conclusion, our results revealed that the three metal oxide NPs (CuO, FeO, and ZnO) significantly improved the morpho-physiological parameters of TBW infected plants and decreased the TBW disease incidence by reshaping the tomato rhizosphere bacterial community. The early increase of tomato rhizosphere microbial community structure improved the diversity and richness of rhizosphere microorganisms, among which CuONPs were the most significant. In summary, NPs (especially CuONPs) have great potential for reducing the occurrence of soil-borne diseases and promoting plant growth and provide a new scientific basis for the prevention and control of soil-borne diseases. However, further field studies are required to elucidate the interaction mechanism of metal oxide NPs rhizosphere soil microbiome.

## Data Availability

The data presented in this study are available within the article.

## Supplementary Materials

The following supporting information can be downloaded at: https://www.mdpi.com/article/10.3390/ijms23010414/s1.
