# Supplementary material for: Effect of the Nanoparticle Exposures on the Tomato Bacterial Wilt Disease Control by Modulating the Rhizosphere Bacterial Community
Source: Int J Mol Sci. 2021 Dec 30;23(1):414. doi: 10.3390/ijms23010414 (PMC8745216; doi:10.3390/ijms23010414)
Supplement: Supplementary file 1 [file ijms-23-00414-s001.zip › ijms-1491113-supplementary.pdf]

Supplementary data

Table S1. Zeta potential (mV) and hydrodynamic diameter of three metal oxide nanoparticle (FeO, CuO and ZnO) suspensions in water.

Analysis	FeONPs	CuONPs	ZnONPs
Zeta potential[a] (mV)	-38.9±0.70	-26.6±0.72	-24.2±0.10
hydrodynamic diameter (nm)	145.07±1.16	95.9±0.26	357.20±0.36

Table S2. Properties of the co-occurrence networks of the bacterial community in rhizosphere soil in the control group and three metal oxide nanoparticle (CuO, FeO and ZnO) group.

Network properties	Second Week				Fourth Week			
	CK	CuO	FeO	ZnO	CK	CuO	FeO	ZnO
**Number of edges**	305	384	226	244	115	117	116	384
**Number of nodes**	105	143	135	125	96	85	100	143
**Modularity**	0.822	0.839	0.935	0.873	0.905	0.91	0.948	0.822
**Average degree**	5.81	6.371	3.348	3.904	2.396	2.753	2.32	2.083
**Positive edges**	168	231	125	166	62	61	61	58
**Negative edges**	137	153	101	78	53	56	55	42

Correlations between bacteria were obtained by the "hmisc" package in R. Spearman's $r > 0.7$ and $<–0.7$ with a *p-value* < 0.01 represented positive and negative correlations, respectively. Other properties of the network were all calculated in Gephi.

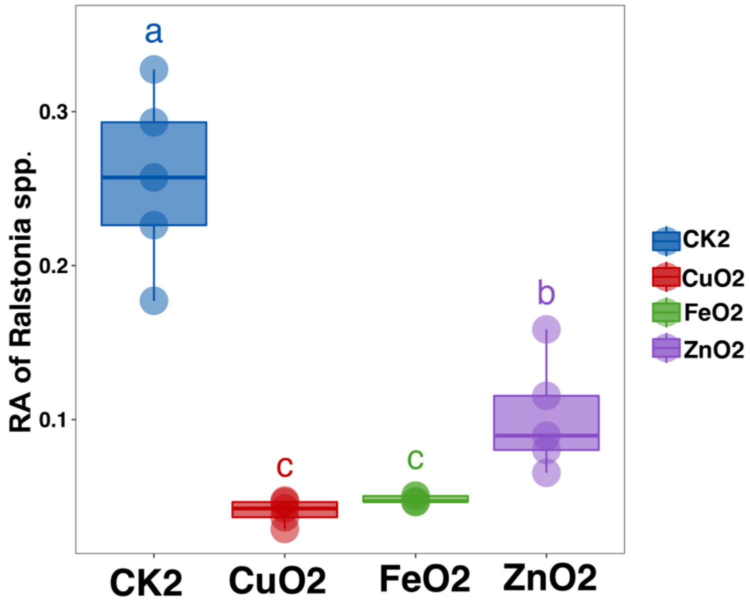

Figure S1. Boxplot of relative abundance of *Ralstonia* spp Abbreviation: 2nd week of nanoparticles treatment (CK2, CuO2, ZnO2 and FeO2)
